# Eosinophilic Granulomatosis with Polyangiitis Presenting as Acute Polyneuropathy Mimicking Guillain-Barre Syndrome

**DOI:** 10.1155/2015/981439

**Published:** 2015-06-23

**Authors:** Carlos R. Camara-Lemarroy, Adrian Infante-Valenzuela, Hector J. Villareal-Montemayor, Carlos A. Soto-Rincon, Javier A. Davila-Olalde, Hector J. Villareal-Velazquez

**Affiliations:** ^1^Servicio de Neurologia, Hospital Universitario “Dr. José E. González” y Facultad de Medicina, Universidad Autónoma de Nuevo León, Madero y Gonzalitos, S/N, 64460 Monterrey, NL, Mexico; ^2^Departamento de Medicina Interna, Hospital Universitario “Dr. José E. González” y Facultad de Medicina, Universidad Autónoma de Nuevo León, Madero y Gonzalitos, S/N, 64460 Monterrey, NL, Mexico

## Abstract

Eosinophilic granulomatosis with polyangiitis (EGPA) is a small-vessel vasculitis associated with antineutrophil cytoplasmic antibodies (ANCAs) which commonly affects the peripheral nervous system. A 38-year-old female with a history of asthma presented with a 2-week history of bilateral lower extremity paresthesias that progressed to symmetric ascending paralysis. Nerve conduction studies could not rule out Guillain-Barre syndrome (GBS) and plasmapheresis was considered. Her blood work revealed marked eosinophilia (>50%), she had purpuric lesions in her legs, and a head magnetic resonance image showed evidence of pansinusitis. Coupled with a history of asthma we suspected EGPA-associated neuropathy and started steroid treatment. The patient showed rapid and significant improvement. ANCAs were later reported positive. ANCA-associated vasculitides present most often as mononeuritis multiplex, but they can mimic GBS and should always be considered in the differential diagnosis, since the treatment strategies for these conditions are radically different.

## 1. Introduction

Churg-Strauss syndrome, a small-vessel vasculitis associated with antineutrophil cytoplasmic antibodies (ANCAs), was first described in 1951 by Churg and Strauss as a rare disease characterized by disseminated necrotizing vasculitis occurring among patients with asthma and eosinophilia. It is now termed eosinophilic granulomatosis with polyangiitis (EGPA). The diagnosis of EGPA is based on ACR 1990 criteria (4 or more of the following: asthma, eosinophilia >10%, neuropathy, nonfixed pulmonary infiltrates, paranasal sinus abnormality, and extravascular eosinophils) [1]. During the vasculitic phase, besides renal and pulmonary disease, central and peripheral nervous system involvement may occur, with polyneuropathy, radiculopathy, and mononeuritis multiplex being common presentations [2]. Guillain-Barre syndrome (GBS) is a clinical syndrome of an acute inflammatory polyneuropathy, characterized by mild sensory loss, ascending weakness, and hypo- or areflexia, progressing to a nadir over up to four weeks. Cerebrospinal fluid evaluation demonstrates albuminocytologic dissociation in 90% of cases. GBS encompasses a number of subtypes with evidence of different clinical, neurophysiological, and immunological mechanisms [3]. In two-thirds there is history of an infection, usually of the upper respiratory system or gastroenteritis. We present a case of EGPA presenting as GBS syndrome. Both GBS and EGPA are rare diseases (approximately 1/100,000), and differentiating between GBS and EGPA-associated vasculitic polyneuropathy is important, considering the different management strategies.

## 2. Case Presentation

Our patient was a 38-year-old female with an 18-year history of asthma. Two weeks before her admission to the emergency department, she developed distal bilateral lower limb paresthesias that rapidly progressed to weakness that prevented her from standing from a chair or walking. On examination the patient was alert and cooperative. She had lower limb weakness (2/5 in both distal and proximal segments, bilaterally), hyporeflexia, and hypoesthesia. She had distal upper limb paresthesias, but strength and reflexes were normal. She had purpuric lesions in the anterior portion of both lower limbs. Oxygen saturation was normal and she did not complain of dyspnea. She had no meningeal signs or fever. Her chest X-ray was normal and the only relevant findings on her routine blood work-up were a marked eosinophilia (13,200, 53%, normal range 0–7%) and an elevated erythrocyte sedimentation rate (49 mm/h: normal range 0–20). Renal function and urine tests were normal. A head MRI showed pansinusitis. Cerebrospinal fluid analysis showed 10 leucocytes and normal glucose and protein. Nerve conduction studies revealed bilateral peroneal, tibial, and sural conduction blocks, with absent F-waves, as well as a left median nerve conduction block. The rest of upper extremity nerves were unaffected. Considering the history of asthma and eosinophilia, we suspected an eosinophilic vasculitic polyneuropathy and immediately started our patient on steroids (1 g methylprednisolone IV). The next day there was a dramatic improvement in the patient's strength (4/5 in both lower limbs) and c-ANCAs were reported positive (1 : 80, normal range <1 : 20). We continued steroid treatment (5 days), added cyclophosphamide (1 g single dose), and cancelled the planned plasmapheresis. Bronchial hyperreactivity was confirmed with pulmonary function tests and a skin biopsy revealed eosinophilic infiltrates. We diagnosed our patient with EGPA-associated polyneuropathy. The patient responded well to treatment, regaining full lower limb strength. She was discharged 10 days later, without any other organ involvement or new symptoms at 3 months of follow-up.

## 3. Discussion

Peripheral neuropathy is quite common in patients with EGPA. Pathological findings of vasculitic neuropathy are characterized by axonal degeneration of nerve fibers caused by vasculitis-induced ischemia. In the largest published series of patients with EGPA, 51.4–60% had peripheral neuropathy at presentation [4, 5]. Mononeuritis multiplex was slightly more common than symmetric polyneuropathy, and the lower limbs were predominately affected. In contrast to lung or renal involvement, peripheral neuropathy alone is rarely life threatening but does significantly affect quality of life. Although less than 40% of patients with EGPA have positive ANCAs, this subgroup of patients presents most often with neuropathy [2, 4]. Our patient was positive for c-ANCA, while the majority (around 80%) of patients are p-ANCA positive, although no differences in clinical characteristics have been found among these subgroups of patients [2, 4]. ANCA positivity has also been associated with lower mortality [2, 4].

There are at least 6 previous cases reported in the literature where EGPA presented as GBS (Table 1). In 1996, Ishiura et al. reported the case of a 74-year-old man who presented with eosinophilic pneumonia and later developed GBS [6]. However, this patient did not meet criteria for EGPA. Ng et al. first reported a case of both clinically and electrophysiologically mimicked GBS, but it was later found to be a case of EGPA due to findings of persistent eosinophilia, positive ANCAs, and eosinophilic vasculitis in a sural nerve biopsy [7]. In 1998, Keven et al. reported the case of a patient diagnosed with GBS who later developed ANCA-positive nephrotic syndrome, suggesting that the polyneuropathy could be secondary to necrotizing vasculitis [8]. Since then, at least 3 other cases of EGPA presenting as GBS have been reported, all with acute ascending polyneuropathy and neurophysiologic studies suggestive of GBS [9–11]. Eosinophilia [10] and ANCA positivity [9, 11] were the factors that led to the correct diagnosis, supported in some cases by treatment failure with IV immunoglobulin [10, 11]. All of these cases followed an aggressive course, with multiorgan involvement and death in 1 case [11], and response only after combination immunosuppressive treatment (corticosteroids and cyclophosphamide) in the other 2 [9, 10].

Although our patient also presented with a symmetric ascending paralysis and hyporeflexia, a history of asthma, severe eosinophilia, and skin purpuric lesions raised our suspicion of EGPA. Compared to the cases mentioned above, our patient was younger, received early treatment with steroids, and had no other organ involvement, and her course was relatively benign. EGPA and other vasculitides should always be part of the differential diagnosis of GBS, as the first line treatments may differ. While steroids are of no use (and may even be harmful) in GBS, they are the mainstay of treatment in EGPA [2]. Adding cyclophosphamide could reduce recurrence of EGPA and may be life-saving when there is multiple organ involvement. On the other hand, IV immunoglobulin and plasmapheresis may be effective in both EGPA and GBS [3].

## Figures and Tables

**Table 1:** Characteristics of patients with EGP presenting as GBS.

Reference	Age (sex)	Clinical manifestations	Laboratory findings	NCS	Other	Treatment	Outcome
[7]	58 M	Symmetric weakness, hyporeflexia, and asthma	Eosinophilia, elevated ERS, and p-ANCA+	Mixed demyelinating polyneuropathy and multifocal absent F-waves	Lung infiltrates	P, CyC, and S	Expired
[8]	57 M	Symmetric weakness and hyporeflexia	p-ANCA+ and elevated ERS	Demyelinating polyneuropathy and multifocal absent F-waves	Nephritis	S	Improved

[9]	51 F	Symmetric weakness, hyporeflexia, asthma, and nasal polyposis	Eosinophilia, elevated CRP, and ANCA−	Motor asymmetric axonal polyneuropathy and multifocal absent F-waves		IvIG and S	Improved

[10]	74 M	Symmetric weakness, hyporeflexia, and asthma	Eosinophilia	Mixed demyelinating polyneuropathy		S and CyC	Improved

[11]	64 F	Asymmetric weakness, hyporeflexia, asthma, diplopia, and sinusitis	Elevated ESR, eosinophilia, and p-ANCA+	Motor axonal and demyelinating polyneuropathy and multifocal absent F-waves	Multiorgan disease	IvIG, CyC, and S	Expired

Present case	36 F	Symmetric weakness, hyporeflexia, asthma, purpura, and pansinusitis	Elevated ESR, eosinophilia, and p-ANCA+	Mixed axonal asymmetric polyneuropathy		S and CyC	Improved

NCS: nerve conduction studies; ESR: erythrocyte sedimentation rate; CRP: C-reactive protein; IvIG: intravenous immunoglobulin; CyC: cyclophosphamide; P: plasmapheresis; S: steroids.
